# Cataract and diabetic retinopathy

**Published:** 2011-09

**Authors:** James Rice

**Affiliations:** Vitreoretinal consultant, University of Cape Town, Division of Ophthalmology, Faculty of Health Sciences, H53 Old Main Building, Groote Schuur Hospital, Observatory 7925, South Africa. Email: james.rice@uct.ac.za

**Figure:**
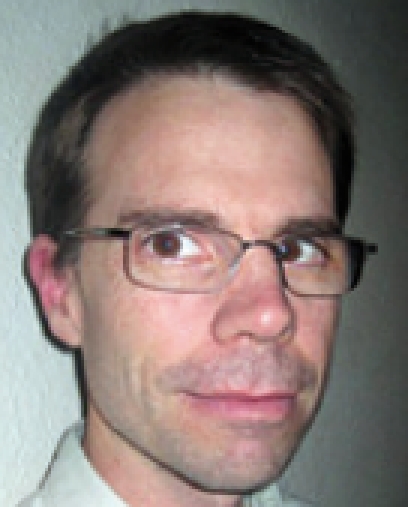


When managing the cataract of a patient with diabetes, you should remember that cataract surgery may make diabetic retinopathy worse. Eyes with mild to moderate non-proliferative diabetic retinopathy at the time of surgery are considered less at risk. Those with severe non-proliferative and proliferative diabetic retinopathy have a higher risk of progressive disease.^1^ Clinically significant macular oedema (CSMO) present at the time of surgery is likely to progress and eyes with previously treated CSMO are at increased risk of recurrence. The risk of progression is increased if the operation is complicated by excessive manipulation, vitreous loss, or severe post-operative inflammation.

Ideally, when the cataract does not preclude laser treatment, you should achieve and maintain effective control of retinopathy and maculopathy for at least three months before surgery.

The severity of the cataract sometimes prevents adequate examination or treatment of the retina in patients with diagnosed or suspected severe non-proliferative and proliferative diabetic retinopathy. In this case, you should deliver pan-retinal photocoagulation either during the procedure or in the early post-operative period. When performing intraoperative pan-retinal photocoagulation with an indirect ophthalmoscope, you should fill the anterior chamber with viscoelastic and place a corneal suture. Complete the pan-retinal photocoagulation before inserting the intraocular lens. This will provide a stable anterior chamber and optimal view, particularly if you anticipate indentation of the periphery.

If you plan to give laser treatment with a contact lens in the early post-operative period, then you should suture the cataract wound. If it is still considered hazardous to use a contact lens then effective slit lamp laser can still be applied through a non-contact 78D or 90D lens. You can also use indirect laser for pan-retinal photocoagulation.

If the patient has diabetic maculopathy and/or more advanced retinopathy, consider intravitreal triamcinolone or anti-VEGF at the end of the procedure to reduce macular oedema. Triamcinolone targets the inflammation that exacerbates the oedema. Anti-VEGFs also reduce retinal swelling and may improve visual outcomes.^2^ Intravitreal steroids may cause raised intraocular pressure and anti-VEGF agents increase the risk of tractional complications in eyes with fibrovascular proliferation. You should still apply macular laser for CSMO post-operatively.

**Figure:**
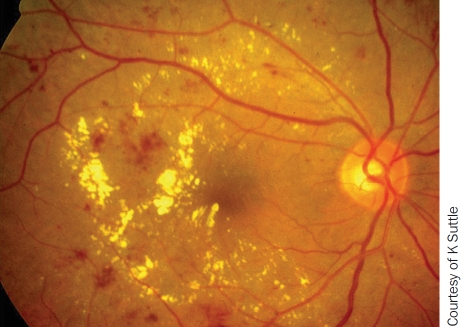
Consider intravitreal triamcinolone or anti-VEGF at the end of surgery to reduce macular oedema

**‘You should remember that cataract surgery may make diabetic retinopathy worse’**

In diabetes patients, it is very important to minimise post-operative inflammation. You should use post-operative topical non-steroidal anti-inflammatory drugs in addition to routine topical steroid preparations, particularly in those with pre-existing macular oedema.^3^

In summary, diabetes patients with mild to moderate diabetic retinopathy and no maculopathy have a good prognosis following cataract surgery. You should treat more advanced retinopathy or maculopathy at least three months prior to surgery if possible. Whereas laser is the most recognised form of treatment, pharmacological agents play an important role in the management of these patients. It is also important to monitor high-risk patients in the post-operative period.
